# Delays in repeat HIV viral load testing for those with elevated viral loads: a national perspective from South Africa

**DOI:** 10.1002/jia2.25542

**Published:** 2020-07-08

**Authors:** Matthew P Fox, Alana T Brennan, Cornelius Nattey, William B MacLeod, Alyssa Harlow, Koleka Mlisana, Mhairi Maskew, Sergio Carmona, Jacob Bor

**Affiliations:** ^1^ Department of Epidemiology Boston University School of Public Health Boston MA USA; ^2^ Department of Global Health Boston University School of Public Health Boston MA USA; ^3^ Health Economics and Epidemiology Research Office, Department of Internal Medicine, School of Clinical Medicine, Faculty of Health Sciences University of the Witwatersrand Johannesburg South Africa; ^4^ National Health Laboratory Service Johannesburg South Africa

**Keywords:** viral load, viral suppression, monitoring, retention, laboratory testing, cohort, HIV

## Abstract

**Introduction:**

In South Africa, HIV patients with an elevated viral load (VL) should receive repeat VL testing after adherence counselling. We set out to use a national HIV Cohort to describe time to repeat viral load testing across South Africa and identify predictors of time to repeat testing.

**Methods:**

We conducted a cohort study of prospectively collected laboratory data. HIV treatment guidelines have changed over time in South Africa, but call for repeat VL testing within six months if 400 to 1000 copies/mL and two to three months if >1000 copies/mL. We included patients with suppressed viral loads (indicating they are on ART) and a first elevated VL (>400 copies/mL) between April 2004 and December 2014. Follow‐up began at first elevated VL and continued until repeat testing, loss to follow‐up or December 2016. We calculated adjusted hazard ratios (aHR) using Cox proportional hazard models.

**Results:**

Of 371,648 patients with a VL > 400, 83.9% (311,790) had a repeat VL, in a median (IQR) of 7.0 (4.1 to 12.2) months. Of those with a first viral load 400 to 1000 copies/mL, 56.4% had a repeat VL within guideline recommended six months (defined as up to nine months), whereas among those >1000 copies/mL only 47.7% had a repeat viral load within guideline recommended two to three months (defined as up to six months). We found a small increase in repeat testing associated with higher VL value (aHR 1.11; 95% CI: 1.10 to 1.12 comparing >1000 vs 400 to 1000 copies/mL) and very low CD4 counts at first elevated VL (aHR 1.16; 95% CI: 1.13 to 1.19 comparing CD4 < 50 vs <500 cells/mm^3^). We also found strong variation in time to repeat VL testing by province.

**Conclusions:**

Median time to repeat viral load testing for those with an elevated viral load was longer than guidelines recommend. Future work should identify whether delays are due to patient or provider factors.

## 1 INTRODUCTION

After patients initiate antiretroviral therapy (ART) for treatment of HIV, laboratory monitoring of patient progress is critical to ensuring treatment success. For individual patients, this means reaching and maintaining a suppressed viral load. At the population level, programme success is often assessed based on the UNAIDS recommended 90‐90‐90 targets [1], of which the third 90 seeks to get 90% of those on ART to achieve viral suppression. Achieving this goal is particularly important as trials [2] and cohort studies [3, 4] have shown that those who are virally suppressed are extremely unlikely to transmit the virus to uninfected partners. This is further supported by observational data showing the reductions in HIV incidence are associated with HIV treatment in Uganda [5] even if trials have yet to show such benefits in large treat‐all programmes [6, 7, 8, 9].

The World Health Organization recommends national programmes which use viral load monitoring for patients on ART [10] to determine whether treatment has been successful and to guide clinical staff on when to switch treatment to second‐line regimens, indicated if the virus is resistant to first‐line therapy. Not all countries are able to provide viral load testing as part of national programmes but for those that do, patients with an elevated viral load are recommended to undergo adherence counselling and then have a repeat measure within three to six months to determine if treatment failure has occurred and the treatment regimen should be switched. In the absence of widespread resistance testing in low‐ and middle‐income countries, patients who do not resuppress after adherence counselling are typically switched to second‐line treatment. While timely repeat testing is important to guide clinical care, the limited data that exist suggests that time to repeat viral load testing varies strongly by country and clinic and retesting is often delayed [11, 12, 13, 14, 15] and this can have negative consequences for patient morbidity, onward transmission and the spread of resistant strains.

Delays in repeat viral load testing are likely related to both patient and provider‐level factors. Patients who have an unsuppressed viral load may not return on time for visits in which counselling and repeat monitoring would be done, or may drop out of care altogether. However, even if patients do follow prescribed clinic visit schedules, providers may not be fully aware of testing protocols or may feel it is safe to allow more time before repeating the viral load in patients who have had a first elevated viral load. Delays could also be related to patient burden and clinic volume. In some settings, clinicians may not even be aware that a patient returning for care had a first elevated viral load due to gaps in clinical record keeping. Additionally, most sites lack clear site level information on their performance at repeat testing, limiting the ability of clinic managers to make changes. Whatever the reasons, the first step towards rectifying the problem is quantifying it and identifying areas where the problem is greatest, so they can be targeted for intervention.

South Africa has used viral load monitoring since the inception of its national programme in 2004. Under national guidelines, patients with an elevated viral load should receive repeat viral load testing after adherence counselling to determine if resuppression has occurred or if regimen change is needed. There have been studies that have explored time to repeat viral load testing among those who are not suppressed in South Africa [11], but they have largely been from single clinics and are often from well resourced, heavily researched sites. To date, no national level perspective on time to repeat viral load testing exists. We set out to use anonymized data from South Africa’s National HIV Laboratory Cohort [16, 17, 18, 19, 20, 21] to describe time to repeat viral load testing across South Africa and identify predictors of time to repeat testing.

## 2 METHODS

### 2.1 Cohort creation

For this analysis, we used deidentified data from a cohort originally created using laboratory data that were linked to create a national HIV cohort. We briefly describe the process of the linkage here to give context for the data used even though this analysis only used deidentified data. More detail is provided elsewhere [22, 23]. The primary data source for this linkage was the database of all viral loads conducted by South Africa’s National Health Laboratory Service (NHLS). NHLS has been the sole provider of laboratory investigations for all of South Africa’s public‐sector HIV treatment programme since ART scale up began in 2004 with the exception of KwaZulu Natal, which began using the NHLS in mid‐2010. NHLS has kept a record of all laboratory investigations in a corporate data warehouse. While the data describe the national cohort of patients accessing HIV care in South Africa, records do not contain a validated unique patient identifier but do contain information on name, date of birth, clinic, sex and date of test for each laboratory, in addition to the laboratory result.

Because each of these fields for individual laboratories can be miscoded, misspelled or left missing, laboratory results were matched using probabilistic matching techniques previously described [16, 17, 19, 20, 23]. Briefly, the Fellegi–Sunter approach [24, 25] was used to compare laboratories on the key variables described above (name, surname, birth date, sex, facility and province) with Jaro–Winkler [25, 26] string comparisons [27, 28]. A weighted average of each of the values was used for comparison. Further linkage was done to account for name inversions and a list of >16,000 nicknames, translated names and common misspellings. Finally a graphical technique was applied to break linkages that appear to be improbable. After linkage, the NHLS database could be analysed as a national cohort of patients enrolled in HIV care since 2004. The cohort was validated against a set of nearly 60,000 laboratory records matched manually by a team of research assistants who found that the approach had 6.3% overmatching (laboratories that were matched should not have been) and 1.4% undermatching (laboratories that should have been matched were not) [22]. The cohort was then deidentified for all further analyses.

### 2.2 Analytic cohort

We included all patients 18 years of age or older at their first suppressed viral load within the deidentified cohort who had at least one suppressed viral load (≤400 copies/mL) at any time point indicating that patients had initiated HIV treatment and achieved viral suppression. We then limited the analytic cohort to those who had an elevated viral load (>400 copies/mL) at some point after their initial suppressed viral load between 1 April 2004 and 31 December 2014. During this time period, treatment protocols called for only CD4 monitoring prior to treatment (until 2016 [29, 30], when CD4 thresholds were removed [31]) followed by viral load testing at ART initiation (until 2009) or at six months on ART (after 2009) then yearly thereafter. As a proxy for time on treatment we subtracted the date of first laboratory result of any kind (an indication of entering care) from the date of eligibility (first elevated viral load after previously being suppressed).

### 2.3 Follow‐up

Follow‐up time started on the date of first elevated viral load after previously being suppressed, until the time of a repeat viral load. We excluded viral loads conducted within two weeks of the first elevated viral load as these were likely confirmatory tests. Follow‐up time accrued from the date of first elevated viral load until the earliest of: 1) a repeat viral load measurement (primary outcome); 2) completion of 24 months of follow‐up (defined as 24 months without a laboratory value [32, 33, 34, 35]); or 3) dataset closure on 31 December 2016. Because this is a laboratory cohort with no visit data, failure to have a repeat viral load test within 24 months is the same as being lost to follow‐up. We did not include anyone with a first elevated viral load after December 2014 to allow each person a potential of 24 months of follow‐up. Unlike most cohorts, we can identify when patients move from one facility to another through linkage of laboratory results, even if the patient self‐transferred. Patients who self‐transfer typically appear as lost to follow‐up in clinical cohorts result in an overestimation in estimates of attrition. We did not censor a patient if they transferred to a new facility, but instead included repeat viral loads if they were within the relevant time period even if subsequent viral load measurements occurred at a different facility.

### 2.4 Outcome

Our primary outcome was time to repeat viral load after a first elevated viral load, after previously being suppressed. Within the HIV care and treatment programme in South Africa, protocols for monitoring patients with an elevated viral load have changed over time. Guidelines have called for adherence counselling for patients with an elevated viral load and have changed over time. Table S1 shows the recommendations over time. Prior to 2019, those with a measurement between 400 and 1000 copies/mL should have a repeat viral load at six months, whereas patients with a viral load >1000 copies/mL should have a repeat viral load test within three months. Thus, we looked at the proportion of patients who received repeat testing consistent with these guideline monitoring timeframes. To give patients sufficient time to get repeat testing we defined appropriate testing to have occurred if a repeat test was done within nine months for those with a viral load between 400 and 1000 copies/mL and within six months for those with a viral load >1000 copies/mL. We note that this is different from other analyses where we have used a stricter definition of repeat testing. As a secondary outcome, we assessed time to suppressed viral load, that is time to a repeat viral load test where the result was <400 copies.

#### 2.4.1 Statistical methods

We described time from first elevated viral load until a repeat viral load using Kaplan–Meier curves. We looked for predictors of time to repeat viral load test using Cox proportional hazards regression. Potential predictors included sex, age, CD4 count at time of elevated viral load (defined as closest CD4 count within +12 months), time on ART, value of elevated viral load (400 to 1000,1001 to 10,000, >10,000 copies/mL), year of elevated viral load, clinic size (total number of patients who had initiated ART at time of elevated viral load, divided into quintiles) and province. We did not use any imputation for missing data.

The study was approved by the Boston University Institutional Review Board and the Human Research Ethics Committee of the University of the Witwatersrand. We only used deidentified data. Both ethics boards approved analysis of deidentified data with a waiver of consent.

## 3 RESULTS

**Figure 1:**
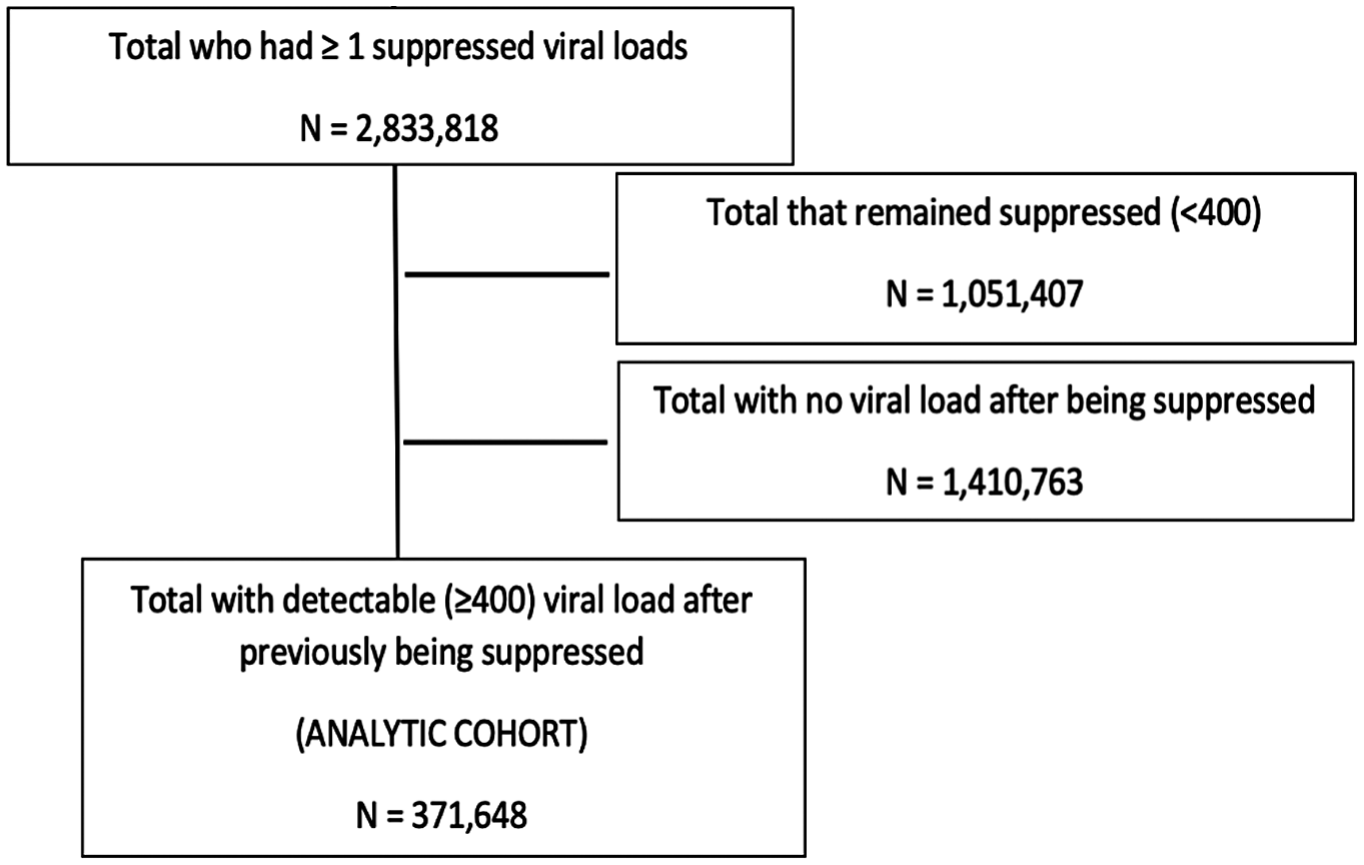
Study eligibility flow chart for creation of a cohort of patients with an initial elevated viral load (≥400 copies/mL) in South Africa’s National HIV Cohort between 2004 and 2014.

Approximately 3.5 million subjects over the age of 18 years old had a viral load measurement done between 1 April 2004 and 31 December 2014 (Figure 1). Of these, 371,648 were eligible for inclusion in the analytic cohort. Patient characteristics at the data of first unsuppressed viral load after first suppressed viral load are summarized in Table 1, stratified by whether or not the patient had a repeat viral load after the enrolment elevated viral load. The majority of the cohort (67%) was female and median (IQR) age was 36 (30 to 43). In total 84% of the cohort had their first elevated viral load between 2010 and = 1 suppressed viral load" : 5/26/2020, 01:21:10 PM" timestamp="1590513670463">2014.

**Table 1:** Characteristics of patients in South Africa’s National HIV Cohort with a first elevated viral load (>400 copies/mL) after an initially suppressed viral load between 2004 and 2014 (n = 371,648)^a^

Characteristic		Repeat viral load (Row %)	No repeat viral load (Row %)	Total (column %)
Sex				
Female	n (%)	210,811 (85.2)	36,542 (14.8)	247,353 (67.4)
Male	n (%)	97,010 (81.1)	22,599 (18.9)	119,609 (32.6)
Age (years)	Median (IQR)	36 (30 to 43)	35 (30 to 43)	36 (30 to 43)
<25	n (%)	17,936 (79.3)	4693 (20.7)	22,629 (6.1)
25 to 30	n (%)	47,695 (83.0)	9804 (17.0)	57,499 (15.5)
30 to 40	n (%)	132,777 (84.6)	24,112 (15.4)	156,889 (42.2)
40 to 50	n (%)	77,052 (85.2)	13,366 (14.8)	90,418 (24.3)
>50	n (%)	36,330 (82.2)	7883 (17.8)	44,213 (11.9)
CD4 count at ART initiation (cell/mL)	Median (IQR)	335 (206 to 497)	277 (149 to 434)	326 (196 to 488)
<50	n (%)	10,790 (69.4)	4,768 (30.6)	15,558 (4.3)
50 to <100	n (%)	14,440 (77.0)	4,318 (23.0)	18,758 (5.2)
100 to <200	n (%)	46,850 (81.4)	10,718 (18.6)	57,568 (16.1)
200 to ‐<350	n (%)	86,806 (84.6)	15,848 (15.4)	102,654 (28.7)
350 to <500	n (%)	68,142 (86.3)	10,836 (13.7)	78,978 (22.1)
≥500	n (%)	74,377 (88.0)	10,166 (12.0)	84,543 (23.6)
First elevated viral load (copies/mL)	Median (IQR)	2574 (810 to 23599)	6448 (1048 to 66950)	2891 (837 to 28728)
400 to <=1000	n (%)	96,773 (86.9)	14,552 (13.1)	111,325 (29.6)
>1000	n (%)	215,017 (82.6)	45,306 (17.4)	260,323 (70.4)
Time since entry into HIV care as proxied by first laboratory result	Median (IQR)	38.4 (23.8 to 57.9)	34.4 (20.0 to 53.7)	37.8 (23.2 to 57.2)
Year of first elevated viral load (eligibility)				
2004/2005	n (%)	556 (83.1)	113 (16.9)	669 (0.2)
2006/2007	n (%)	10,253 (85.5)	1740 (14.5)	11,993 (3.2)
2008/2009	n (%)	38,317 (85.2)	6658 (14.8)	44,975 (12.1)
2010/2011	n (%)	80,147 (84.6)	14,626 (15.4)	94,773 (25.5)
2012/2013	n (%)	111,522 (83.2)	22,479 (16.8)	134,001 (36.1)
2014	n (%)	70,995 (83.3)	14,242 (16.7)	85,237 (22.9)
Province				
Eastern Cape	n (%)	36,419 (81.6)	8224 (18.4)	44,643 (12.0)
Free State	n (%)	16,331 (82.6)	3430 (17.4)	19,761 (5.3)
Gauteng	n (%)	84,006 (83.9)	16,109 (16.1)	100,115 (26.9)
Kwazulu‐Natal	n (%)	60,499 (87.5)	8605 (12.5)	69,104 (18.6)
Limpopo	n (%)	25,432 (82.4)	5420 (17.6)	30,852 (8.3)
Mpumalanga	n (%)	31,080 (82.9)	6373 (17.1)	37,453 (10.1)
North West	n (%)	26,751 (81.9)	5900 (18.1)	32,651 (8.8)
Northern Cape	n (%)	5931 (81.8)	1321 (18.2)	7252 (2.0)
Western Cape	n (%)	25,341 (84.9)	4476 (15.1)	29,817 (8.0)

^a^ First two columns are row percentages, total column is column percentage.

We found that, overall, 83.9% (95% Confidence Interval (CI): 83.7% to 84.0%) had a repeat viral load test within 24 months of the first elevated viral load. Median (interquartile range (IQR)) time to repeat viral load testing, as estimated in Kaplan–Meier failure curves, was 7.5  months (5.3 to 12.0) among patients with first viral load 400 to 1000 copies and 6.2 months (4.0 to 11.1), among patients with a first elevated viral load >1000 copies (Table 2). At first elevated viral load, the median (IQR) viral load was 2891 copies/mL (837 to 28,728) and the median (IQR) CD4 count was 326 cells/mm^3^ (196 to 488)). Of those who had an elevated viral load between 400 and 1000 copies/mL, 86.9% (n = 96,773) had repeat viral load within 24 months, but only 56.4% (n = 54,552) had a repeat viral load within the recommended six months (defined as up to nine months). Of those who had an elevated viral load >1000 copies/mL, 82.6% (n = 215,017) had repeat viral load, but only 47.7% (n = 102,502) had a repeat viral load within the recommended two to three months (defined as up to six months).

**Table 2:** Repeat viral load testing and repeat testing within guidelines among those with an initial elevated viral load (>400 copies/mL) in South Africa’s National HIV Cohort between 2004 and 2014 (n = 371,648)

First elevated viral load (copies/mL)	Follow‐up time median (IQR)	Time to repeat viral load among those with a repeat viral load median (IQR)	N (%) repeat viral load within 24 months	N (%) repeat viral load within guidelines^a^
400 to 1000	7.5 (4.7 to 12.4)	7.5 (5.3 to 12.0)	96,773 (86.9)	54,552 (56.4)
>1000	6.2 (3.2 to 11.9)	6.2 (4.0 to 11.1)	215,017(82.6)	102,502 (47.7)
Total	6.5 (3.7 to 12.0)	6.5 (4.0 to 11.7)	311,790 (83.9)	157,054 (50.7)

LTF, loss to follow‐up; VL, viral load.

^a^ Within two to ‐three months for those with a first elevated viral load >1000 (operationalized as within six months) and within six months for those with a first elevated viral load 400 to 1000 (operationalized as within nine months).

Although 84% of the cohort had a repeat VL test after entering care, 52.3% of the cohort was virally suppressed at that test. Median (IQR) follow‐up time to first suppressed viral load after first elevated viral load among the cohort was 12.9 months (6.7 to 26.8).

**Figure 2:**
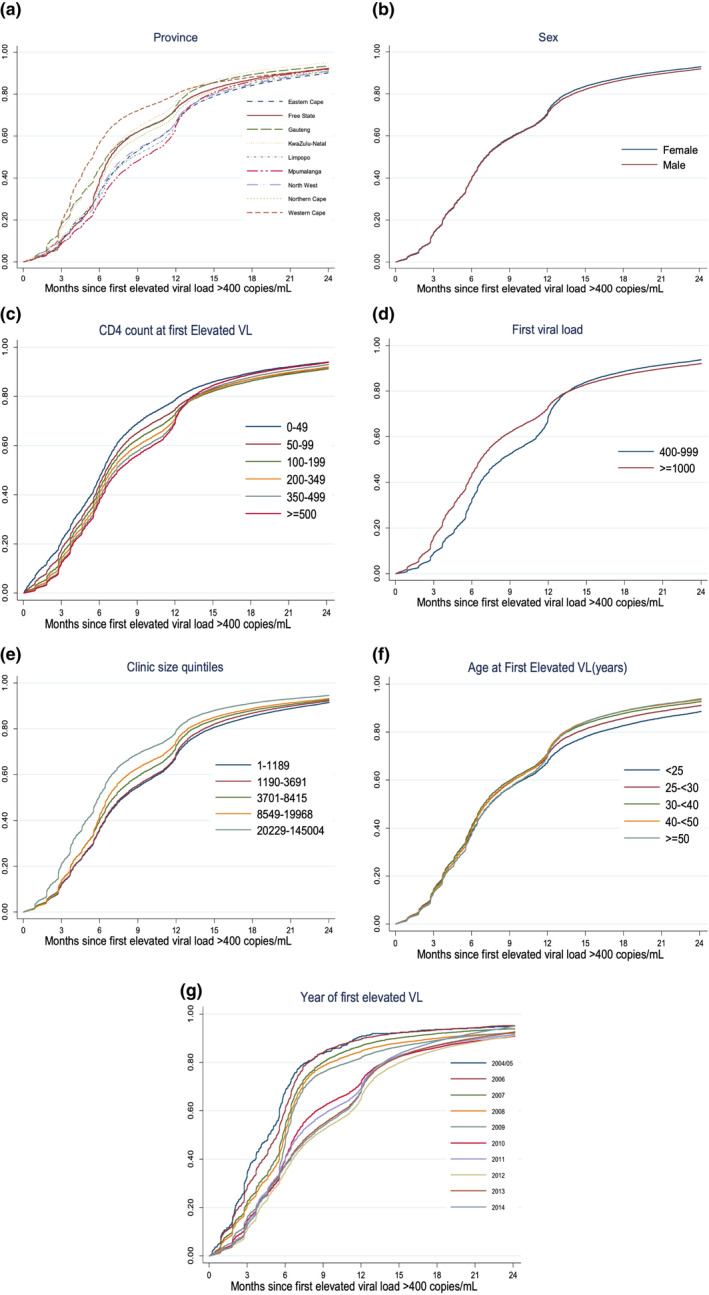
Kaplan–Meier curves of time to repeat viral load testing among those with an initial elevated viral load (>400 copies/mL) in South Africa’s National HIV Cohort between 2004 and 2014 (n = 371,648) stratified by predictors: **(A) Province**; **(B) Sex**; **(C) CD4 count**; **(D) Viral Load (E) Clinic size**; **(F) Age**; **(G) Year of eligibility**.

Stratified Kaplan–Meier curves of time to repeat viral load testing are shown in Figure 2 and adjusted hazard ratios for repeat viral load testing are shown in Table 3. We found a small increase in likelihood of repeat testing associated with both higher viral load value (adjusted Hazard Ratio (aHR) 1.11; 95% CI: 1.10 to 1.12 comparing viral load >1000 vs 400 to 1000 copies/mL) and very low CD4 counts at first elevated viral load (aHR 1.16; 95% CI: 1.13 to 1.19 comparing CD4 < 50 vs >=500 cells/mm^3^). Young age was associated with longer time until repeat testing (<25 vs >=50 years old, aHR: 0.83; 95% CI: 0.81 to 0.84). Larger clinic size was associated with increased repeat testing (highest quartile vs lowest aHR: 1.28; 95% CI: 1.26 to 1.29). We also found that province and year of eligibility were predictive of time to repeat viral load, but little association with sex or year at elevated viral load. The median time to repeat viral load testing increased year on year from 2005 until 2012 when it appears to have levelled off at a median of about seven months (Table 4). However, the per cent with a repeat viral load within guidelines has declined from 77% in 2005 to 45% in 2014.

**Table 3:** Hazard Ratios of Predictors of Time to Repeat Viral Load among those with an initial elevated viral load (>400 copies/mL) in South Africa’s National HIV Cohort between 2004 and 2014 (n = 371,648)

Factor	N	Repeat viral load	Person‐months (Total)	Rate/100 person months	Adjusted hazard ratio (95% CI)
CD4 count at first elevated viral load (cells/mL)					
<50	15,558	10,790	92,753.5	11.70	1.16 (1.13 to 1.19)

50 to 100	18,758	14,440	138,522.2	10.47	1.01 (0.99 to 1.03)
100 to 200	57,568	46,850	470,351.8	10.01	0.97 (0.95 to 0.98)
200 to 350	102,654	86,806	879,715.2	9.92	0.97 (0.95 to 0.98)
350 to 500	78,978	68,142	689,931.4	9.92	0.98 (0.97 to 0.99)
≥500	84,543	74,377	742,412.4	10.02	Reference
Age at first elevated viral load (years)					
<25	22,629	17,936	202,928.4	8.87	0.83 (0.81 to 0.84)

25 to 30	57,499	47,695	496,770.8	9.65	0.91 (0.89 to 0.92)
30 to 40	156,889	132,777	1,318,031.7	10.13	0.95 (0.94 to 0.97)
40 to 50	90,418	77,052	758,057.4	10.22	1.00 (0.98 to 1.01)
>50	44,213	36,330	360,535.4	10.08	Reference
Sex					
Male	119,609	97,010	995,448.8	9.80	0.93 (0.93 to 0.94)
Female	247,353	210,811	2,093,344.2	10.07	Reference
Year of eligibility					
2004/2005	669	556	3,487.2	16.03	1.60 (1.47 to 1.74)
2006/2007	11,993	10,253	74,478.2	13.83	1.47 (1.43 to 1.50)
2008/2009	44,975	38,317	326,519.1	11.79	1.27 (1.25 to 1.29)
2010/2011	94,773	80,147	823,232.7	9.78	0.97 (0.96 to 0.98)
2012/2013	134,001	111,522	1,197,656.5	9.36	0.91 (0.88 to 0.93)
2014	85,237	70,995	709,105.7	10.01	Reference
Clinic size quintile (patients)					
1 to 1189	105,673	87,240	945,011.6	9.23	Reference
1190 to 3691	82,068	68,543	725,460.4	9.50	1.00 (0.99 to 1.01)
3701 to 8415	67,779	57,128	571,252.4	10.05	1.04 (1.03 to 1.05)
8549 to 19968	60,716	51,239	493,025.8	10.45	1.11 (1.10 to 1.12)
20229 to 145004	55,412	47,640	398,481.1	12.02	1.28 (1.26 to 1.29)
Years on ART at elevated viral load					
<1	24,863	18,836	174,251.3	10.80	Reference
1 to 1.9	73,068	60,013	579,968.7	10.34	1.02 (1.00 to 1.04)
2 to 2.9	75,844	63,638	629,302.9	10.11	1.03 (1.01 to 1.05)
3 to 3.9	65,929	56,035	562,833.4	9.96	1.03 (1.01 to 1.05)
4 to 4.9	47,843	40,818	424,211.9	9.62	1.02 (1.01 to 1.04)
5+	84,101	72,450	751,146.1	9.64	1.05 (1.03 to 1.07)
Elevated viral load (copies/mL)					
400 to 1000	110,205	95,793	1,006,914.2	9.51	Reference
>1000	261,443	215,997	2,114,800.1	10.21	1.11 (1.10 to 1.12)
Province					
EC	44,643	36,419	416,501.9	8.79	1.03 (1.02 to 1.05)
FS	19,761	16,331	166,910.1	9.84	1.18 (1.15 to 1.20)
GP	100,115	84,006	781,089.0	10.76	1.24 (1.22 to 1.26)
KZN	69,104	60,499	542,008.7	11.22	1.45 (1.43 to 1.47)
MP	37,453	31,080	355,095.7	8.80	1.01 (0.99 to 1.03)
NW	32,651	26,751	299,726.5	8.97	1.01 (0.99 to 1.03)
NC	7252	5931	62,774.0	9.50	1.15 (1.11 to 1.19)
WC	29,817	25,341	219,101.4	11.63	1.44 (1.42 to 1.47)
LP	30,852	25,432	290,891.2	8.79	Reference

**Table 4:** Relationship between year of first elevated viral load (>400 copies/mL) and time to repeat viral load testing in South Africa’s National HIV Cohort between 2004 and 2014 (n = 371,648)

Year of first elevated viral load	Follow‐up months Median (IQR)	% Repeat viral load	% With repeat viral load within guidelines^a^
2005	3.7 (1.8 to 6.3)	83.1	77.3
2006	4.7 (2.0 to 6.7)	85.6	70.6
2007	5.5 (2.7 to 7.3)	85.4	64.0
2008	5.8 (2.8 to 8.1)	83.8	63.7
2009	6.0 (3.7 to 8.1)	85.8	64.0
2010	6.4 (3.7 to 12.0)	84.5	53.6
2011	6.5 (3.9 to 12.2)	84.7	51.6
2012	7.3 (3.9 to 12.8)	83.0	44.8
2013	6.9 (3.7 to 12.3)	83.4	46.7
2014	6.8 (3.4 to 12.2)	83.3	45.3

CI, confidence interval; EC, Eastern Cape; FS, Free State; GP, Gauteng; KZN, KwaZulu Natal; LP, Limpopo; MP, Mpumalanga; NC, Northern Cape; NW, North West; VL, viral load; WC, Western Cape.

^a^ Within two to three months for those with a first elevated viral load >1000 (operationalized as within six months) and within six months for those with a first elevated viral load 400 to 1000 (operationalized as within nine months).

We found variation in the proportion of patients having a repeat viral load and the median time to repeat viral load testing by province. Table 5 shows the variation by province. At the province level, the median (IQR) time to repeat testing was 5.1 months (3.1 to 9.2) in Western Cape but 8.2 months (4.6 to 12.9) in Mpumalanga province.

**Table 5:** Geographical variation in median time to repeat viral load testing among those with an initial elevated viral load (>400 copies/mL) in South Africa’s National HIV Cohort between 2004 and 2014 (n = 371,648)

Province	N	Median (IQR)	% Repeat viral load	% Repeat viral load within guidelines^a^
Eastern Cape	44,643	7.4 (4.0 to 12.9)	81.6	43.2
Free State	19,761	6.4 (4.0 to 11.9)	82.6	50.6
Gauteng	100,115	6.0 (3.0 to 11.8)	83.9	54.8
KwaZulu‐Natal	69,104	6.2 (3.5 to 11.5)	87.6	54.6
Limpopo	30,852	7.5 (4.7 to 12.7)	82.4	41.3
Mpumalanga	37,453	8.2 (4.6 to 12.9)	83.0	38.9
North West	32,651	7.1 (4.1 to 12.7)	81.9	43.3
Northern Cape	7252	6.6 (3.9 to 12.2)	81.8	48.6
Western Cape	29,817	5.1 (3.1 to 9.2)	84.9	66.8

^a^ Within two to three months for those with a first elevated viral load >1000 (operationalized as within six months) and within six months for those with a first elevated viral load 400 to 1000 (operationalized as within nine months).

## 4 DISCUSSION

In the first national analysis of time to repeat viral load testing among South African HIV patients with an elevated viral load (i.e. indicated to have repeat testing after adherence counselling), we found that most patients (about 84%) received a repeat viral load, but only half did so within the time periods called for by guidelines. There was variation in the time to repeat testing by province, with provincial level estimates ranging from a median of 5.1 months to 8.2 months. While eight months (the upper end of the distribution we found) is higher than desired, for a national programme this is not surprising. What may be more surprising was the lower end where the best performing province had a median of about five months to repeat testing.

Of interest, there was little difference between the time to repeat viral load testing among those who had an elevated viral load between 400 and 1000 copies/mL and those with a viral load >1000 copies/mL. While we saw a small, but precise, difference, it would be a mistake to over‐interpret these differences given the large size of the database (giving narrow confidence intervals) and the fact that our estimates surely have some bias related to the imprecise matching algorithm used to create longitudinal patient records. Those with viral loads >1000 copies/mL were slightly more likely to have any repeat test, which would be expected given that those with higher viral loads are recommended to have repeat testing earlier. Despite these findings, we found that fewer patients were receiving repeat testing within the recommended time frames of within six months for those with a viral load 400 to 1000 copies/mL and within two to three months for those with a viral load >1000 copies/mL. We found that only about half of patients with an elevated viral load met these guidelines, emphasizing the need for renewed efforts to ensure timely testing. Of note, South Africa’s 2019 ART Clinical Guidelines [36] has remove the distinction between a viral load 400 to 1000 and >1000, requiring all patients with a viral load >50 to have repeat testing after three months. It remains unclear what impact this will have on repeat testing given the current limitations on repeat testing at three months for those with high viral loads, but monitoring of patterns of repeat testing with more recent data will be important for improving programmatic outcomes.

The reasons for delays in repeat testing have not yet been well described and more work is needed to understand what the causes of these delays are in order to inform effective interventions to reduce time to repeat testing. Our data cannot shed light on this problem because we neither have patient level visit data nor information on reasons for delays. Still, we suspect that the reasons relate to both patient and provider level factors. For patients, the burden of accessing treatment combined with HIV‐related stigma may lead to delays in returning for clinic visits and specialized programmes may be needed to counsel patients who are having difficulty resuppressing [37]. On the other hand if providers are the main driver, either through lack of familiarity with guidelines, a belief that it is safe to wait to retest patients or because clinics are overburdened and cannot accommodate the volume of patients they must care for, then interventions targeted at providers are likely to have more benefit [38, 39].

While the delays in repeat testing we found were longer than hoped, they are in line with what has been observed in other published studies. In South Africa, a median of between roughly 2.9 months in cohort of patients in nine clinics largely in Gauteng province [11] and 6.8 months in KwaZulu Natal province [13] has been observed. Thus our estimate of a median of six months is within the range of what has previously been observed, but provides a much more representative picture of repeat testing within South Africa because of the national scope of our analysis. This may also suggest that some of the single clinic analyses of repeat testing come from well‐resourced clinics (certainly the one we are affiliated with [11] is) and therefore may not be representative of the national programme. On the positive side, we found that most patients did in fact have repeat testing. As neither of the studies we identified that reported time to repeat testing reported the percentage of patients who had repeat testing, we cannot compare our results to other studies, but our results suggest that the need for repeat testing is understood even if the timing of repeat testing is longer than desirable.

The delays we observed in repeat testing are particularly problematic given attempts to reach 90‐90‐90 targets and to reduce transmission. Further delays in switching have been shown to be associated with poorer outcomes [11, 40] and increase the risk of transmission. Perhaps, even more concerning is the fact that the time to repeat viral load testing has been increasing over time. To an extent this is expected, as programmatic scale up would likely make it more difficult to manage the large number of patient in care and the time to repeat testing does appear to have stabilized in the later years of the cohort. Further efforts to reduce the time to repeat testing may need to focus on those areas with this longest delays to make the most impact.

Our study is the largest to date to assess time to repeat viral load testing among patients with an elevated viral load and one of the first to able to account for silent transfers. However, our study also has some important limitations. First, our cohort is created from a laboratory database with no unique identifier. The probabilistic matching cohort can lead to both over matching (where we link records that should not be linked) and undermatching (where we fail to link records that should be linked). Since we use laboratory records to identify repeat testing in this analysis, overmatching could create the appearance of faster repeat testing for some patients. It could also make it appear that some patients who never had repeat testing did have a repeat viral load. Undermatching would likely cause us to overestimate time to repeat testing and to overestimate failure to have a repeat test. As we do not know the exact mix of over and undermatching in this subset of the cohort, we cannot say in which direction this would likely bias our results, though we believe undermatching is more common than overmatching. Second, we do not have clinic visit data in this cohort, only laboratory data. While this would not impact our ability to determine the time to repeat testing, visit data could tell us if delays were related to patient missing visits or not. Third, while our data represent a national profile from the largest HIV programme in the world, it is still only one programme that is unique within the region and the results cannot be extrapolated to other countries.

## 5 CONCLUSIONS

In conclusion, we found that repeat testing for patients with an elevated viral load was longer than recommended by current treatment guidelines, but that the proportion of patients receiving repeat testing was high. In order to achieve the UNAIDS 90‐90‐90 targets, a renewed effort at ensuring patients with an elevated viral load receive care that will allow them to resuppress in as short a time as possible is essential. Future work should specifically address the reasons for delays in repeat testing in order to ensure targeted interventions are most likely to succeed.

## COMPETING INTERESTS

The authors declare they have no competing interests.

## AUTHORS’ CONTRIBUTIONS

MPF designed the study, conceived the analyses and wrote the first draft of the manuscript. ATB and CN conducted the analyses. WBM, MM and JB developed the analytic cohort, advised on the analyses and edited the manuscript. AH, KM and SC provided input into the analyses and edited the manuscript. All authors have read and approved the final manuscript.

### 1 FUNDING

This work was supported by grants 1R01AI115979‐01 and 1K01MH105320‐01A1 from the National Institutes of Health. The funder had no role in study design, data collection and analysis, decision to publish, or preparation of the manuscript. WBM and SC were supported by USAID through Cooperative Agreement AID‐674‐A‐12‐0020 (WM) from the United States Agency for International Development (USAID). USAID played no role in the manuscript, did not review the manuscript and played no role in the decision to submit.

## Supporting information


**Table S1.** South African national treatment guidelines for repeat viral load testing

## References

[1] UNAIDS . 90‐90‐90 An ambitious treatment target to help end the AIDS epidemic. Geneva, Switzerland: UNAIDS; 2014.

[2] Cohen MS , Chen YQ , McCauley M , Gamble T , Hosseinipour MC , Kumarasamy N , et al. Prevention of HIV‐1 infection with early antiretroviral therapy. N Engl J Med. 2011;365(6):493–505. 21767103 10.1056/NEJMoa1105243 PMC3200068

[3] Bavinton BR , Pinto AN , Phanuphak N , Grinsztejn B , Prestage GP , Zablotska‐Manos IB , et al. Viral suppression and HIV transmission in serodiscordant male couples: an international, prospective, observational, cohort study. Lancet HIV. 2018;5(8):e438–47. 30025681 10.1016/S2352-3018(18)30132-2

[4] Rodger AJ , Cambiano V , Bruun T , Vernazza P , Collins S , van Lunzen J , et al. Sexual activity without condoms and risk of HIV transmission in serodifferent couples when the HIV‐positive partner is using suppressive antiretroviral therapy. JAMA. 2016;316(2):171. 27404185 10.1001/jama.2016.5148

[5] Grabowski MK , Serwadda DM , Gray RH , Nakigozi G , Kigozi G , Kagaayi J , et al. HIV Prevention efforts and incidence of HIV in Uganda. N Engl J Med. 2017;377(22):2154–66. 29171817 10.1056/NEJMoa1702150 PMC5627523

[6] Iwuji CC , Orne‐Gliemann J , Larmarange J , Balestre E , Thiebaut R , Tanser F , et al. Universal test and treat and the HIV epidemic in rural South Africa: a phase 4, open‐label, community cluster randomised trial. Lancet HIV. 2018;5(3):e116–25. 29199100 10.1016/S2352-3018(17)30205-9

[7] Havlir DV , Balzer LB , Charlebois ED , Clark TD , Kwarisiima D , Ayieko J , et al. HIV testing and treatment with the use of a community health approach in Rural Africa. N Engl J Med. 2019;381(3):219–29. 31314966 10.1056/NEJMoa1809866 PMC6748325

[8] Hayes RJ , Donnell D , Floyd S , Mandla N , Bwalya J , Sabapathy K , et al. Effect of universal testing and treatment on HIV incidence ‐ HPTN 071 (PopART). N Engl J Med. 2019;381(3):207–18. 31314965 10.1056/NEJMoa1814556 PMC6587177

[9] Makhema J , Wirth KE , Pretorius Holme M , Gaolathe T , Mmalane M , Kadima E , et al. Universal testing, expanded treatment, and incidence of HIV infection in Botswana. N Engl J Med. 2019;381(3):230–42. 31314967 10.1056/NEJMoa1812281 PMC6800102

[10] World Health Organization (WHO) . Consolidated guidelines on the use of antiretroviral drugs for treating and preventing HIV infection recommendations for a public health approach. 2nd ed. Geneva, Switzerland: WHO; 2016. 27466667

[11] Rohr JK , Ive P , Horsburgh CR , Berhanu R , Shearer K , Maskew M , et al. Marginal structural models to assess delays in second‐line HIV treatment initiation in South Africa. De Socio GV, ed. PLoS One. 2016;11:e0161469. 27548695 10.1371/journal.pone.0161469 PMC4993510

[12] Petersen ML , Tran L , Geng EH , Reynolds SJ , Kambugu A , Wood R , et al. Delayed switch of antiretroviral therapy after virologic failure associated with elevated mortality among HIV‐infected adults in Africa. AIDS. 2014;28(14):2097–107. 24977440 10.1097/QAD.0000000000000349 PMC4317283

[13] Narainsamy D , Mahomed S . Delays in switching patients onto second‐line antiretroviral treatment at a public hospital in eThekwini, KwaZulu‐Natal. South Afr J HIV Med. 2017;18(1):a696. 10.4102/sajhivmed.v18i1.696 PMC5843064 29568632

[14] Ramadhani HO , Bartlett JA , Thielman NM , Pence BW , Kimani SM , Maro VP , et al. The effect of switching to second‐line antiretroviral therapy on the risk of opportunistic infections among patients infected with human immunodeficiency virus in Northern Tanzania. Open Forum Infect Dis. 2016;3(1):ofw018. 26949717 10.1093/ofid/ofw018 PMC4776054

[15] Murphy RA , Court R , Maartens G , Sunpath H . Second‐line antiretroviral therapy in sub‐Saharan Africa: it is time to mind the gaps. AIDS Res Hum Retroviruses. 2017;33(12):1181–4. 28793781 10.1089/aid.2017.0134 PMC5709698

[16] Fox MP , Bor J , Brennan AT , MacLeod WB , Maskew M , Stevens WS , et al. Estimating retention in HIV care accounting for patient transfers: a national laboratory cohort study in South Africa. PLoS Med. 2018;15:e1002589. 29889844 10.1371/journal.pmed.1002589 PMC5995345

[17] Carmona S , Bor J , Nattey C , Maughan‐Brown B , Maskew M , Fox MP , et al. Persistent high burden of advanced HIV disease among patients seeking care in South Africa’s national HIV program: data from a nationwide laboratory cohort. Clin Infect Dis. 2018;66:S111–7. 29514238 10.1093/cid/ciy045 PMC5850436

[18] Bor J , Fox MP , Rosen S , Venkataramani A , Tanser F , Pillay D , et al. Treatment eligibility and retention in clinical HIV care: a regression discontinuity study in South Africa. PLoS Med. 2017;14:e1002463. 29182641 10.1371/journal.pmed.1002463 PMC5705070

[19] Nattey C , Maughan‐Brown B , MacLeod W , Maskew M , Carmona S , Fox MP , et al.. Rising CD4 counts at clinical presentation: evidence from a novel national database in South Africa. In: 21st International AIDS Conference. Durban, South Africa; 2016.

[20] Maskew M , Bor J , Macleod W , Carmona S , Sherman G , Fox MP . The youth treatment bulge in South Africa: increasing numbers, inferior outcomes among adolescents on ART. In: 21st International AIDS Conference. Durban, South Africa; 2016.

[21] Bor J , Maskew M , Stevens W , Carmona S , MacLeod W . District prevalence of unsuppressed HIV in South African women: monitoring programme performance and progress towards 90‐90‐90. In: 21st International AIDS Conference. Durban, South Africa (TUAC0205); 2016.

[22] Bor J , Brennan A , Fox MP , Maskew M , Stevens W , Carmona S,, et al. Building a national HIV cohort from routine laboratory data: probabilistic record‐linkage with graphs. bioRxiv. 2018.

[23] Maskew M , Bor J , Hendrickson C , MacLeod W , Bärnighausen T , Pillay D , et al. Imputing HIV treatment start dates from routine laboratory data in South Africa: a validation study. BMC Health Serv Res. 2017;17(1):41. 28095905 10.1186/s12913-016-1940-2 PMC5240407

[24] Fellegi I , Sunter A . A theory for record linkage. J Am Stat Assoc. 1969;40:1183–210.

[25] Jaro MA . Probabilistic linkage of large public health data files. Stat Med. 1995;14(5–7):491–8. 7792443 10.1002/sim.4780140510

[26] Winkler W . Comparator metrics and enhanced decision rules in the fellegi‐sunter model of record linkage. Proc Sect Surv Res. Methods. 1990;354–9.

[27] Herzog TN , Scheuren FJ , Winkler WE . Data quality and record linkage techniques. New York, NY: Springer; 2007.

[28] Christen P . Data matching: concepts and techniques for record linkage, entity resolution, and duplicate detection. New York, NY: Springer; 2012.

[29] South African National Department of Health . National antiretroviral treatment guidelines. Pretoria, South Africa: South African National Department of Health; 2004.

[30] National Department of Health Republic of South Africa . The South African antiretroviral treatment guidelines. South Africa: National Department of Health Republic of South Africa; 2013.

[31] South African National Department of Health and South African National AIDS Council . Circular on implementation of the universal test and treat strategy for HIV positive patients and differentiated care for stable patients.

[32] Fox MP , Larson B , Rosen S . Defining retention and attrition in pre‐antiretroviral HIV care: proposals based on experience in Africa. Trop Med Int Heal. 2012;17(10):1235–44. 10.1111/j.1365-3156.2012.03055.x PMC3726560 22863075

[33] Chi BH , Yiannoutsos CT , Westfall AO , Newman Jamie E , Zhou Jialun , Cesar Carina , et al. Universal definition of loss to follow‐up in HIV treatment programs: a statistical analysis of 111 facilities in Africa, Asia, and Latin America. PLoS Med. 2011;8:e1001111. 22039357 10.1371/journal.pmed.1001111 PMC3201937

[34] Grimsrud AT , Cornell M , Egger M , Boulle A , Myer L . Impact of definitions of loss to follow‐up (LTFU) in antiretroviral therapy program evaluation: variation in the definition can have an appreciable impact on estimated proportions of LTFU. J Clin Epidemiol. 2013;66:1006–13. 23774112 10.1016/j.jclinepi.2013.03.013 PMC3759810

[35] Kaplan SR , Oosthuizen C , Stinson K , Little F , Euvrard J , Schomaker M , et al. Contemporary disengagement from antiretroviral therapy in Khayelitsha, South Africa: a cohort study. Newell M‐L, ed. PLoS Med. 2017;14:e1002407. 29112692 10.1371/journal.pmed.1002407 PMC5675399

[36] South African National Department of Health . 2019 ART clinical guidelines for the management of HIV in adults, pregnancy, Adolescents, Infants and Neonates. Pretoria, South Africa: South African National Department of Health; 2019.

[37] Katz IT , Ryu AE , Onuegbu AG , Psaros C , Weiser SD , Bangsberg DR , et al. Impact of HIV‐related stigma on treatment adherence: systematic review and meta‐synthesis. J Int AIDS Soc. 2013;16 3 Suppl 2:18640. 24242258 10.7448/IAS.16.3.18640 PMC3833107

[38] Roberts T , Cohn J , Bonner K , Hargreaves S . Scale‐up of routine viral load testing in resource‐poor settings: current and future implementation challenges. Clin Infect Dis. 2016;62(8):1043–8. 26743094 10.1093/cid/ciw001 PMC4803106

[39] Lecher S , Williams J , Fonjungo PN , Kim AA , Ellenberger D , Zhang G , et al. Progress with scale‐up of HIV viral load monitoring — seven sub‐Saharan African Countries, January 2015–June 2016. MMWR Morb Mortal Wkly Rep. 2016;65(47):1332–5. 27906910 10.15585/mmwr.mm6547a2

[40] Levison JH , Orrell C , Losina E , Lu Z , Freedberg KA , Wood R . Early outcomes and the virological effect of delayed treatment switching to second‐line therapy in an antiretroviral roll‐out programme in South Africa. Antivir Ther. 2011;16(6):853–61. 21900717 10.3851/IMP1819 PMC3225051

