# CORRIGENDUM

**DOI:** 10.1002/jia2.25646

**Published:** 2020-12-05

In the article [1], there were errors in the Methods section (2.1 Cohort creation) on e25542.

The last sentence reads:

"The cohort was validated against a set of nearly 60,000 laboratory records matched manually by a team of research assistants who found that the approach had 6.3% overmatching (laboratories that were matched should not have been) and 1.4% undermatching (laboratories that should have been matched were not) [22]."

The last sentence should have read:

"The cohort was validated against a set of nearly 60,000 laboratory records matched manually by a team of research assistants who found that the approach had 1.4% overmatching (laboratory records that were matched should not have been) and 6.3% undermatching (laboratory records that should have been matched were not) [22]."
