# Supplementary material for: Delays in repeat HIV viral load testing for those with elevated viral loads: a national perspective from South Africa
Source: J Int AIDS Soc. 2020 Jul 8;23(7):e25542. doi: 10.1002/jia2.25542 (PMC7343337; doi:10.1002/jia2.25542)
Supplement: Supplementary file 1 — Table S1. South African national treatment guidelines for repeat viral load testing [file JIA2-23-e25542-s001.docx]

**Table S1 - South African National Treatment Guidelines for Repeat Viral Load Testing**

| Year | Guidelines |
| --- | --- |
| 2004 | - 400-5000 copies/mm^3^, repeat viral load in 6 months - > 5000 copies/mm^3^, repeat viral load in 3 months |
| 2010 | - 400-1000 copies/mm^3^, repeat viral load at 6 months - >1000 copies/mm^3^ repeat viral load in 3 months |
| 2013 | - 400-1000 copies/mm^3^, repeat viral load at 6 months - >1000 copies/mm^3^, repeat viral load in 2 months |
